# RE: A step-by-step guide for remote working in the NHS: evaluation of a virtual consultant psychiatrist hiring scheme

**DOI:** 10.1192/bjb.2025.10145

**Published:** 2025-10

**Authors:** Nyembezi Faith Ndebele, Ian McCafferty, Megan Havard

**Affiliations:** Virtual Consultant Psychiatrist, Hampshire and Isle of Wight Healthcare NHS Foundation Trust, Southampton, UK; Consultant Psychiatrist and Deputy Chief Medical Officer, Hampshire and Isle of Wight Healthcare NHS Foundation Trust, Southampton, UK; Foundation Doctor, North Bristol NHS Trust, Bristol, UK

We appreciate Mann-Wineberg and Tomar’s (2025)^1^ interest and thoughtful response to our article.^2^ Our experience post-pandemic of working virtually to provide remote care over a period of three and a half years (ongoing) and delivering over three-thousand virtual appointments has been successful. It has reduced the burden on onsite colleagues and, we hope, also enhanced well-being for all. More importantly, it has enabled delivery of high-quality care to patients. Our model of service delivery relies on a blended approach to care while offering choice to patients. The group of onsite consultants and virtual consultants in our setting have developed into a cohesive, engaged body working collaboratively while supporting each other to provide safe patient care.

Assessments by virtual consultant psychiatrists in our adult mental health service and older persons mental health are largely carried out via secure videoconferencing platforms designed for patient care. Consultants visually observe the patient and carry out full assessments, including collateral histories from family members, carers and others. Prescribing is done electronically. All patients, including those seen by consultants face to face in physical space, have access to a well-being clinic where physical examinations and investigations can be conducted.

Virtual consultants are clinical and educational supervisors, supporting training of resident doctors and teaching of medical students. When required, resident doctors working with virtual consultants carry out physical examinations as part of their usual duties. Supervision is undertaken by virtual consultants as required to support both training needs and service delivery. Virtual consultants in our setting see all patients allocated to them and carry out psychiatric assessments, and there are no instances of patients not being seen who require it. Non-medical staff are part of the multidisciplinary team and contribute to patient care in their different roles. Your response quotes findings mainly from a quality improvement project^3^ during the Covid pandemic involving 37 patients seen in a hybrid memory clinic with a consultant contributing to the assessment by seeing patients remotely. We note the largely positive feedback from patients from this initiative.

Most adults including older adults in the UK have access to mobile devices;^4^ those with care responsibilities as well as patients with neurodevelopmental conditions have welcomed having the option to attend virtually. We aim to be inclusive in our service delivery and accommodate the preferences of our patients and ensure equipment is available to facilitate appointments for those who choose virtual care but may not have access to mobile devices.

We continue to explore the appropriate use of regulated technology in our setting and have started piloting the use of artificial intelligence in the form of ambient voice technology,^5^ which lends itself well to virtual working. Ambient artificial intelligence scribes have shown promise in reducing the workload of clinicians by assisting with documentation and enhancing clinician–patient encounters.^6^

We once again thank the authors for their engagement and hope that this response contributes to productive conversation on this evolving and important topic.
